# Elasticity and Stability of Clathrate Hydrate: Role of Guest Molecule Motions

**DOI:** 10.1038/s41598-017-01369-0

**Published:** 2017-05-02

**Authors:** Jihui Jia, Yunfeng Liang, Takeshi Tsuji, Sumihiko Murata, Toshifumi Matsuoka

**Affiliations:** 1 Environment and Resource System Engineering, Kyoto University, Kyoto, 615-8540 Japan; 2 International Institute for Carbon-Neutral Energy Research (I2CNER), Kyushu University, Fukuoka, 819-0395 Japan; 3 Center for Engineering, Research into Artifacts (RACE), the University of Tokyo, Chiba, 277-8568 Japan; 4 Department of Earth Resources Engineering, Kyushu University, Fukuoka, 819-0395 Japan; 5 Fukada Geological Institute, Tokyo, 113-0021 Japan

## Abstract

Molecular dynamic simulations were performed to determine the elastic constants of carbon dioxide (CO_2_) and methane (CH_4_) hydrates at one hundred pressure–temperature data points, respectively. The conditions represent marine sediments and permafrost zones where gas hydrates occur. The shear modulus and Young’s modulus of the CO_2_ hydrate increase anomalously with increasing temperature, whereas those of the CH_4_ hydrate decrease regularly with increase in temperature. We ascribe this anomaly to the kinetic behavior of the linear CO_2_ molecule, especially those in the small cages. The cavity space of the cage limits free rotational motion of the CO_2_ molecule at low temperature. With increase in temperature, the CO_2_ molecule can rotate easily, and enhance the stability and rigidity of the CO_2_ hydrate. Our work provides a key database for the elastic properties of gas hydrates, and molecular insights into stability changes of CO_2_ hydrate from high temperature of ~5 °C to low decomposition temperature of ~−150 °C.

## Introduction

**Figure 1:**
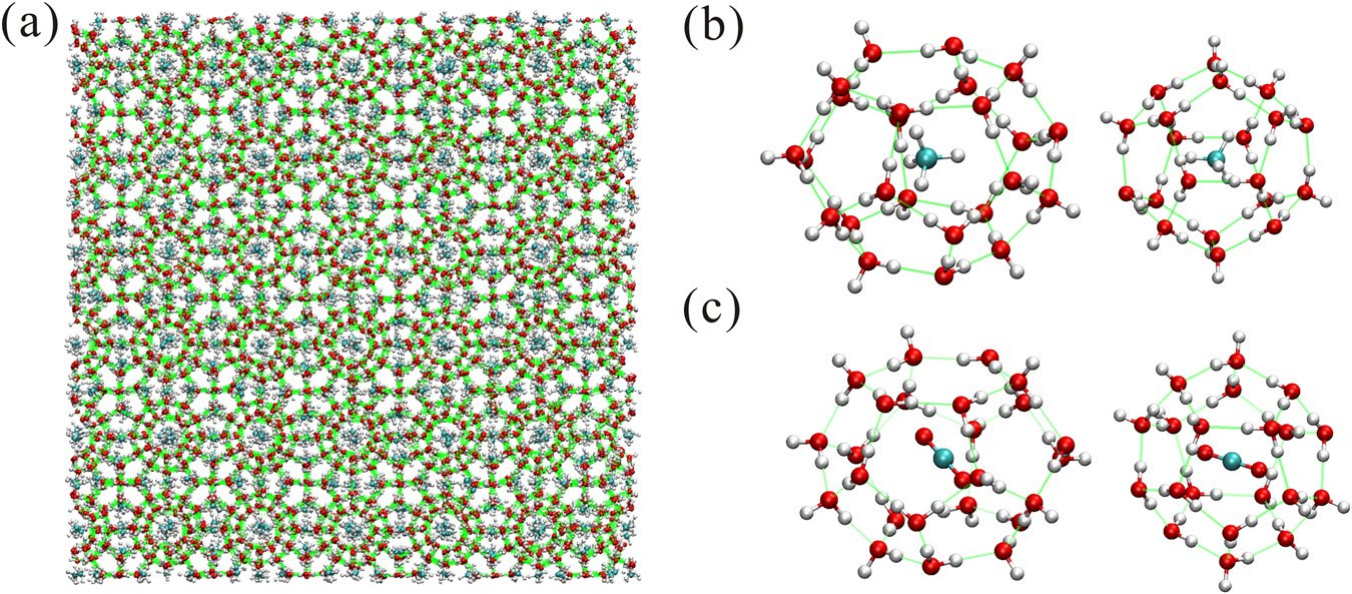
Gas hydrate (sI) microstructure. (**a**) Bulk structure of gas hydrate. (**b**) Large cage (5^12^6^2^) and small cage (5^12^) of CH_4_ hydrate. (**c**) Large cage (5^12^6^2^) and small cage (5^12^) of CO_2_ hydrate. Red, cyan, and white balls represent oxygen, carbon and hydrogen atoms respectively. Green lines represent hydrogen bonds.

Gas hydrate is an ice-like, nonstoichiometric compound that consists of hydrogen-bonded water molecules that form cavities in which “guest” molecules of an appropriate size are contained (Fig. 1(a)). Hydrate forms when free gas and aqueous phases coexist under low temperature and high pressure conditions in deep marine sediments and permafrost zones. Typical “guest” molecules include methane (CH_4_) (Fig. 1(b)) and carbon dioxide (CO_2_) (Fig. 1(c)), which usually form a type-I clathrate structure (sI) with two types of cages: large tetrakaidecahedral 5^12^6^2^ cages (L-cage) and small dodecahedron 5^12^ cages (S-cage)^1, 2^. Gas hydrates occur abundantly in nature and are considered to be future energy resource^2–5^. They have numerous applications including hydrogen storage, natural gas transportation, and gas separation^2, 3, 6–8^. Furthermore, the research on hydrate is crucial to flow assurance^9, 10^ and environmental change^11^. It is also important for astrophysics since CO_2_ and CH_4_ hydrates are likely to occur in Mars^12^ as well as outer solar systems^13^.

The replacement of CH_4_ by CO_2_ in gas hydrate-bearing sediments has received great attention^14–22^ since it may enable long-term storage of CO_2_, which could mitigate the influence of global warming and ocean acidification, and facilitate CH_4_ recovery as a potential future energy resource. Remarkably, CO_2_ hydrates have been clearly observed on the seafloor^23^, implying they can be stable enough even occurring in extremely shallow marine sediments. An understanding of the elastic properties of gas hydrate-bearing sediments is important not only for monitoring their distributions (e.g. via seismic analysis) when CO_2_ replaces CH_4_ from gas hydrate deposits, but also for predicting the stability of their formations^24^. CO_2_ and CH_4_ hydrates have been synthesized in the laboratory^25, 26^. Brillouin scattering experiments^27^, compressional, and shear-wave speed measurements^28^ have been reported for CH_4_ hydrate. However, the mechanical properties of CO_2_ hydrate have rarely been reported, and limited information exists on the differences in mechanical properties between CO_2_ and CH_4_ hydrates. This stimulated our interest to investigate the mechanical properties of CO_2_ hydrate in comparison with CH_4_ hydrate.

CO_2_ hydrate has been studied extensively experimentally in the laboratory in terms of phase change^29^, stability^12, 30–32^, structure^26, 30, 33–37^, cage occupancy^26, 33–37^, and dynamics^38, 39^. X-Ray, neutron diffraction, and dissociation experiments have been used to determine the occupancy of CO_2_. It was found that the occupancy is a function of the synthesis pressure^26, 33–37^. In general, higher overpressure ratio (to the phase equilibrium pressure) during synthesis produced higher occupancies^26, 31^. It reaches full occupancy, when the overpressure ratio is greater than 6^26, 31^. For the hydrate synthesized at the proximity of phase equilibrium pressure, the refined model show that CO_2_ occupied almost all (>99%) of the large cages and roughly 2/3 of small cages^33, 35–37^. NMR measurements^38^ and Raman spectra^39^ have been used to describe the motions of guest molecules with increase in temperature. Clearly, the thermal effect is critical to CO_2_ hydrate^37–39^. CO_2_ hydrate will decompose at either too high or too low temperatures^12, 30–32^. It is the only hydrate known to have a stability limit at low temperatures of approximately −150 °C^12, 30–32^. This is because the vapor pressure of dry ice is lower than the dissociation pressure of the CO_2_ hydrate^12^. However, there lacks of understanding for low-temperature stability limit from a mechanical point of view. For instance, the shear modulus of the CO_2_ hydrate may decrease as the temperature decreases, no information on this aspect has been documented yet.

Molecular dynamics (MD) simulations can provide insights on hydrates at the molecular level with linkages to macroscopic phenomena^17–19, 22, 40–50^, where interactions between guest and host are of particular importance. Those simulation studies provided valuable information on free energy changes for CO_2_ replacing CH_4_ from gas hydrate^17–19^, structural changes induced by various guest molecules^41^, dissociation^42^, nucleation^43, 44^, thermal conductivity^41, 45^, mechanical properties^46–49^, and NMR spectra^50^. Our previous work^46^ has studied the mechanical strength, and explained the underlining mechanisms of strain hardening of CH_4_ hydrate as against normal ice. The compressibility (i.e. the inverse of bulk modulus) has been determined on the basis of the fluctuation theorem for both CH_4_ and CO_2_ hydrates^22^. The elastic moduli of CH_4_ hydrate have also been determined at 0 K by first-principles calculations^49^ and at finite temperatures by classical MD simulations^48^. However, there is no applicable data on shear modulus and elastic constants for CO_2_ hydrate. We have performed MD simulations to construct pressure (P)–temperature (T) diagrams of the elastic properties of CO_2_ hydrate (with full and partial occupancy), CH_4_ hydrate (full occupancy), and hypothetical empty-cage hydrate at 100 different pressure and temperature conditions; this equates to 2,000 different non-equilibrium MD simulations for constant strain-rate deformation tests (Fig. S1). The diagrams cover a wide range of P–T conditions that represent deep marine sediments and permafrost zones. The aim of the work is to obtain *a priori* knowledge on the elastic properties of two different gas hydrates for the purpose of monitoring their distributions in the field during CO_2_ replacement of CH_4_ from gas hydrate deposits.

## Results

### Diagram of elastic constants and elastic moduli

**Figure 2:**
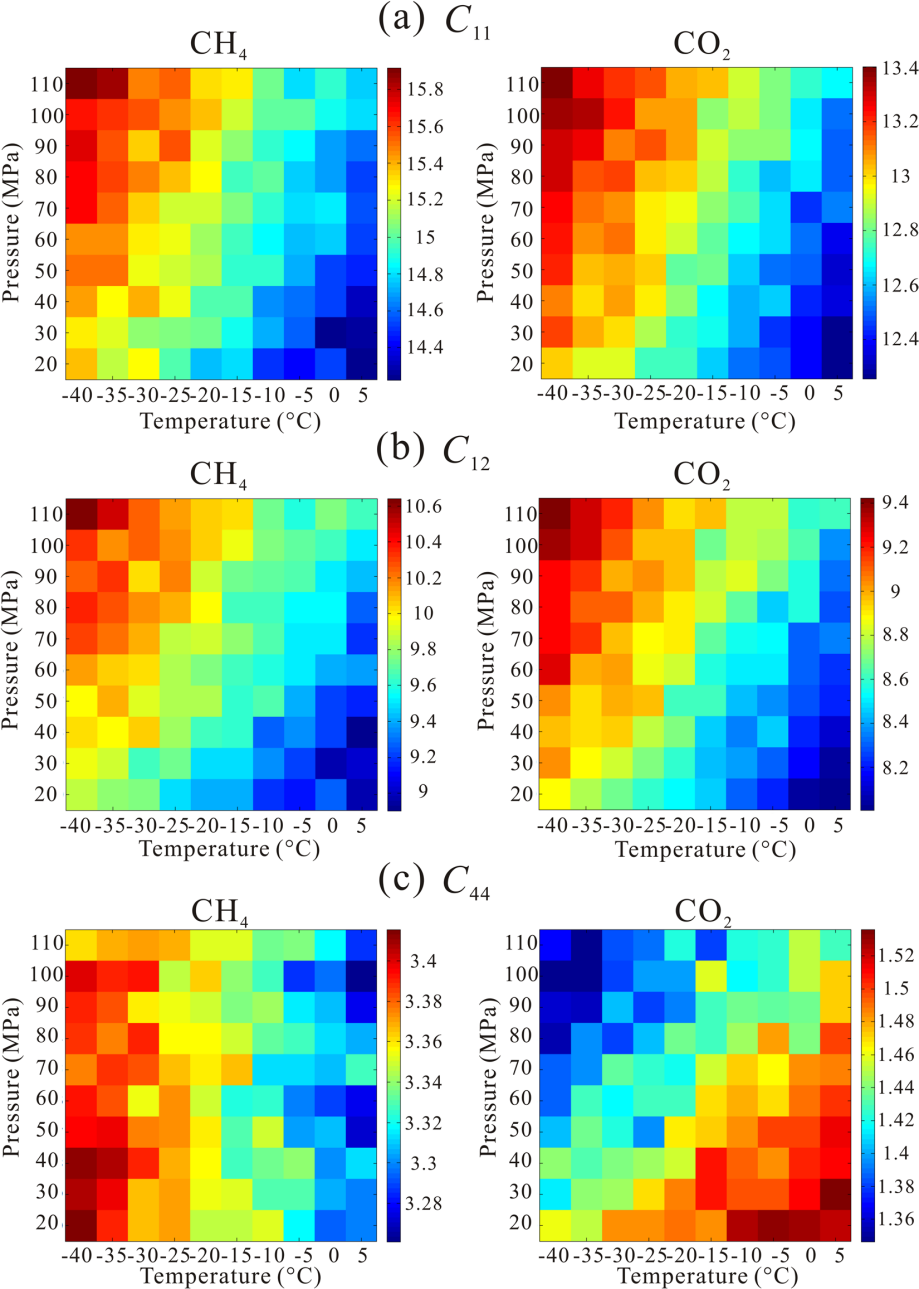
Three independently evaluated elastic constants of CH_4_ and CO_2_ hydrate with full occupancy from −40 °C to 5 °C and 20 MPa to 110 MPa. Red represents large value of elastic constant while blue represents small value with unit of GPa.

At first, we compared the lattice constants for both hydrates. Tables SI and SII present the data under 0.1 MPa, which are in good agreement with previous experimental^37^ and simulation results^41^. For CH_4_ hydrate, the deviation from experiments is less than 0.3%. For CO_2_ hydrate, we found that the estimated lattice constant is very close to the experimental one (the deviation is within 0.3%) if their occupancy is assumed to be the same (ca. 2/3 occupancy of small cages)^37^. Interestingly, we could reproduce that CO_2_ hydrate has smaller lattice constants than CH_4_ hydrate at low temperatures and larger lattice constants than CH_4_ hydrate at high temperatures with a crossover at ~−73.15 °C (200 K). Gas hydrate is a cubic system, which can be characterized by three independent elastic constants *C*_11_, *C*_12_, and *C*_44_. We calculated them by the stress-strain linear relationship as shown in Fig. S1. A fairly good agreement was found between our computed elastic constants of CH_4_ hydrate (*C*_11_ = 13.9, *C*_12_ = 8.6, and *C*_44_ = 3.2) and those from Brillouin scattering measurements (*C*_11_ = 11.9, *C*_12_ = 6.0, and *C*_44_ = 3.4)^27^ at room temperature (22.85 °C and 20 MPa). We constructed P–T diagrams of the elastic constants and elastic moduli of the CH_4_ and CO_2_ hydrates. Figure 2 shows our calculated elastic constants of CH_4_ and CO_2_ hydrates with full occupancy from −40 °C to 5 °C in increments of 5 °C and from 20 MPa to 110 MPa in increments of 10 MPa. The low and high temperatures on the diagrams represent the permafrost zones in the Arctic area and deep marine sediments under the seafloor, respectively. All the elastic constants of the CH_4_ hydrate are comparatively larger than those of the CO_2_ hydrate. *C*_44_ of the CH_4_ hydrate is nearly two times as large as that of the CO_2_ hydrate. Variations of *C*_11_ and *C*_12_ for CO_2_ and CH_4_ hydrates exhibit the same trend, that is, the calculated values decrease with increase in temperature and increase with increase in pressure. It is interesting to note that *C*_44_ exhibits different trends for the two different hydrates: *C*_44_ for the CH_4_ decreases with increase in temperature and appears to be not dependent on pressure. In contrast, *C*_44_ for the CO_2_ hydrate increases with increase in temperature and decreases when the pressure increases.

**Figure 3:**
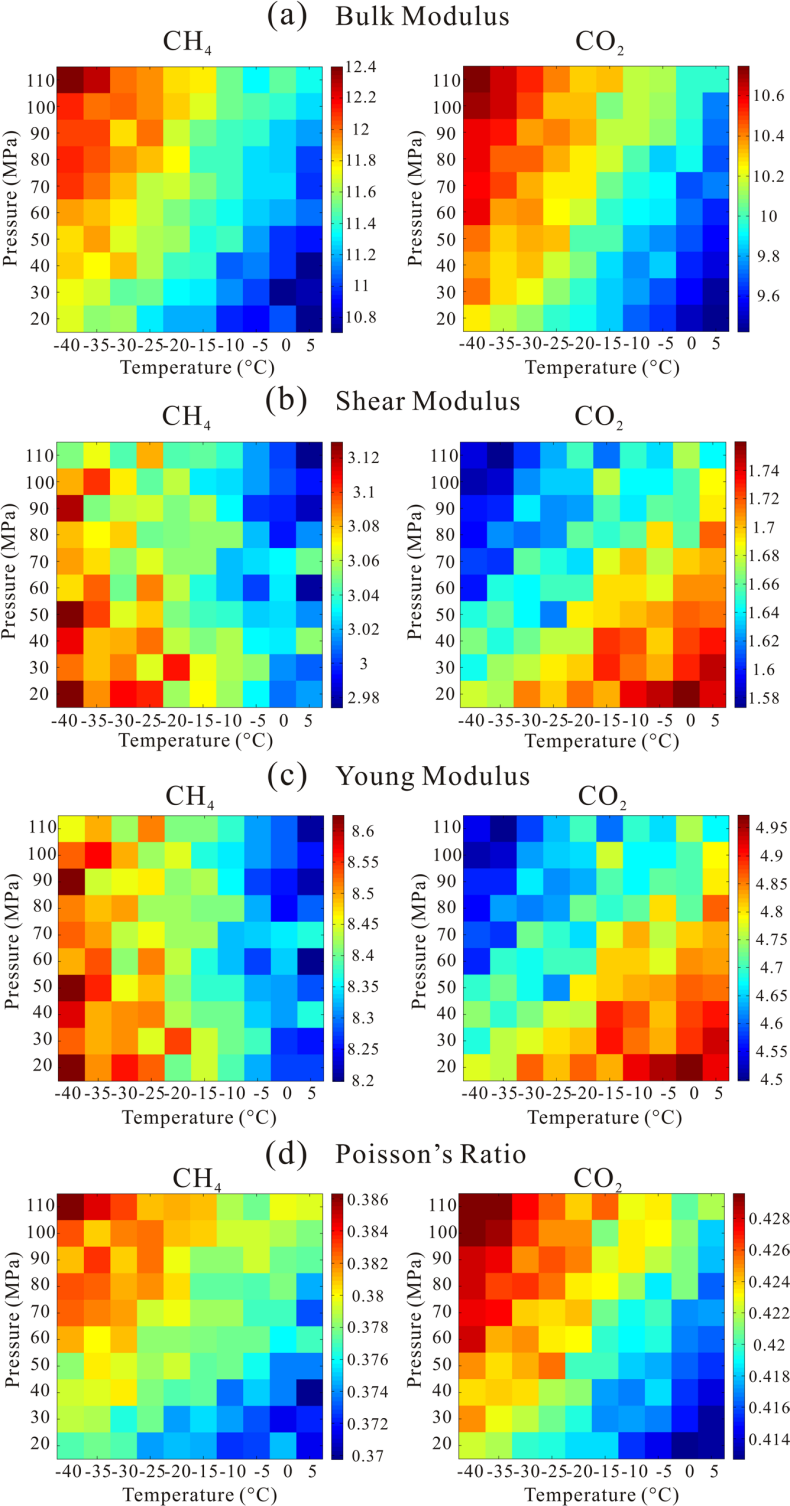
Diagrams of bulk modulus, shear modulus, Young’s modulus, and Poisson’s ratio of CH_4_ and CO_2_ hydrates with full occupancy from −40 °C to 5 °C and 20 MPa to 110 MPa. Red represents large value of elastic moduli while blue represents small value with unit of GPa. Poisson’s ratio has no unit.

Based on the results in Fig. 2, the elastic moduli of the hydrate aggregate were evaluated using the Voigt–Reuss–Hill (VRH) model^51–53^ shown in Fig. 3. The four elastic moduli can be divided into two groups according to different patterns. The first group is composed of the bulk modulus and Poisson’s ratio, which increase with increase in pressure, and decrease with increase in temperature. The trend is as same as that of *C*_11_ and *C*_12_. The bulk modulus of the CH_4_ hydrate is larger than that of the CO_2_ hydrate, which implies that it is harder to compress; this result is consistent with those of previous research calculated using the fluctuation theorem^22^. In contrast, the CH_4_ hydrate has a smaller Poisson’s ratio compared with the CO_2_ hydrate, which indicates less transferred deformation in the lateral direction perpendicular to the applied load. The second group includes shear modulus and Young’s modulus. The trends coincide with those of *C*_44_. The computed results of the CH_4_ hydrate are nearly two times as large as those of the CO_2_ hydrate. The computed P-wave velocity of CH_4_ hydrate is about 0.8 km/s larger than that of CO_2_ hydrate which is consistent with experimental result^21^, whereas the computed S-wave velocity of CH_4_ hydrate is about 0.6 km/s larger than that of CO_2_ hydrate. The shear modulus and Young’s modulus signify material rigidity, which indicates that the CO_2_ hydrate becomes more rigid when the temperature increases. Different potential models (refer to method section and Fig. S2) were employed for CO_2_ hydrate, the same conclusion is reached. It is an interesting and anomalous phenomenon, which has rarely been reported with regard to the crystalline materials. While this finding is on the basis of VRH model^51–53^, it holds for both Voigt and Reuss models, respectively. We hypothesize that this phenomenon is related to the kinetic behavior of the CO_2_ molecules that reside in the cages since the only difference between the two materials is induced by the “guest” molecules. The CO_2_ hydrate micro-structure in Fig. 1(c) shows that if the rotation of the guest CO_2_ molecules is constrained and “locked” in a certain orientation (at low temperature), the elastic properties (related to angle variables of the crystalline cell) of this compound may present anomalous behavior. In contrast, the CH_4_ molecule is more isotropic in shape, the rotational motion of the guest CH_4_ molecules should not be “locked”, and the mechanical properties of the whole structure may present a regular behavior of crystalline materials.

### Rotational motion of CO_2_ and mechanisms for thermal-enhanced stability

We show here that the different properties of CO_2_ and CH_4_ hydrates are attributed to the enclathrated “guest” molecules, which have limited translational motion but substantially more rotation and rattling ability within the cavities^37–39, 41, 50^. In such a case, the linear CO_2_ with comparatively large diameter would result in certain trajectories facilitating its rotation^37–39, 41, 50^. This rotational motion has been attributed to temperature-dependent NMR line-shape anisotropy^50^ and “anomalous” large thermal expansion of CO_2_ hydrate^41^. The primitive unit cell of sI structure includes six L-cages, which are oblate in shape with equatorial planes (parallel to hexagonal faces), and two S-cages without equatorial planes. In the oblate L-cage of the gas hydrate sI, the effective cage radius is smaller in the direction of the cage axis of symmetry (“polar” direction) than that in the equatorial plane. This kind of structure provides the CO_2_ molecule with greater freedom in the equatorial plane than in the “polar” direction perpendicular to this plane. Therefore, the CO_2_ molecule can rotate non-uniformly towards the short symmetry axis inside the L-cage. To characterize the CO_2_ rotational motion, the Cartesian coordinates of oxygen atoms of each step as predicted by MD simulations were output and used to calculate rotational correlation coefficients using a first order Legendre polynomial of the angle between oxygen–carbon–oxygen vectors of the CO_2_ molecules and principal axes expressed by $\cos \,\theta =\mathop{{{\boldsymbol{e}}}_{1}}\limits^{\longrightarrow}\cdot \mathop{{{\boldsymbol{e}}}_{2}}\limits^{\longrightarrow}/|\mathop{{{\boldsymbol{e}}}_{1}}\limits^{\longrightarrow}||\mathop{{{\boldsymbol{e}}}_{2}}\limits^{\longrightarrow}|$ where $\mathop{{{\boldsymbol{e}}}_{1}}\limits^{\longrightarrow}$ denotes the X, Y, and Z axes, $\mathop{{{\boldsymbol{e}}}_{2}}\limits^{\longrightarrow}$ means vectors of the CO_2_ molecules at each step successively during the simulation processes, *θ* is the angle between vectors $\mathop{{{\boldsymbol{e}}}_{1}}\limits^{\longrightarrow}$ and $\mathop{{{\boldsymbol{e}}}_{2}}\limits^{\longrightarrow}$, the dot sign means the dot product, and $||\,\,$ signifies the length of the vectors on a two-dimensional Euclidean space.

**Figure 4:**
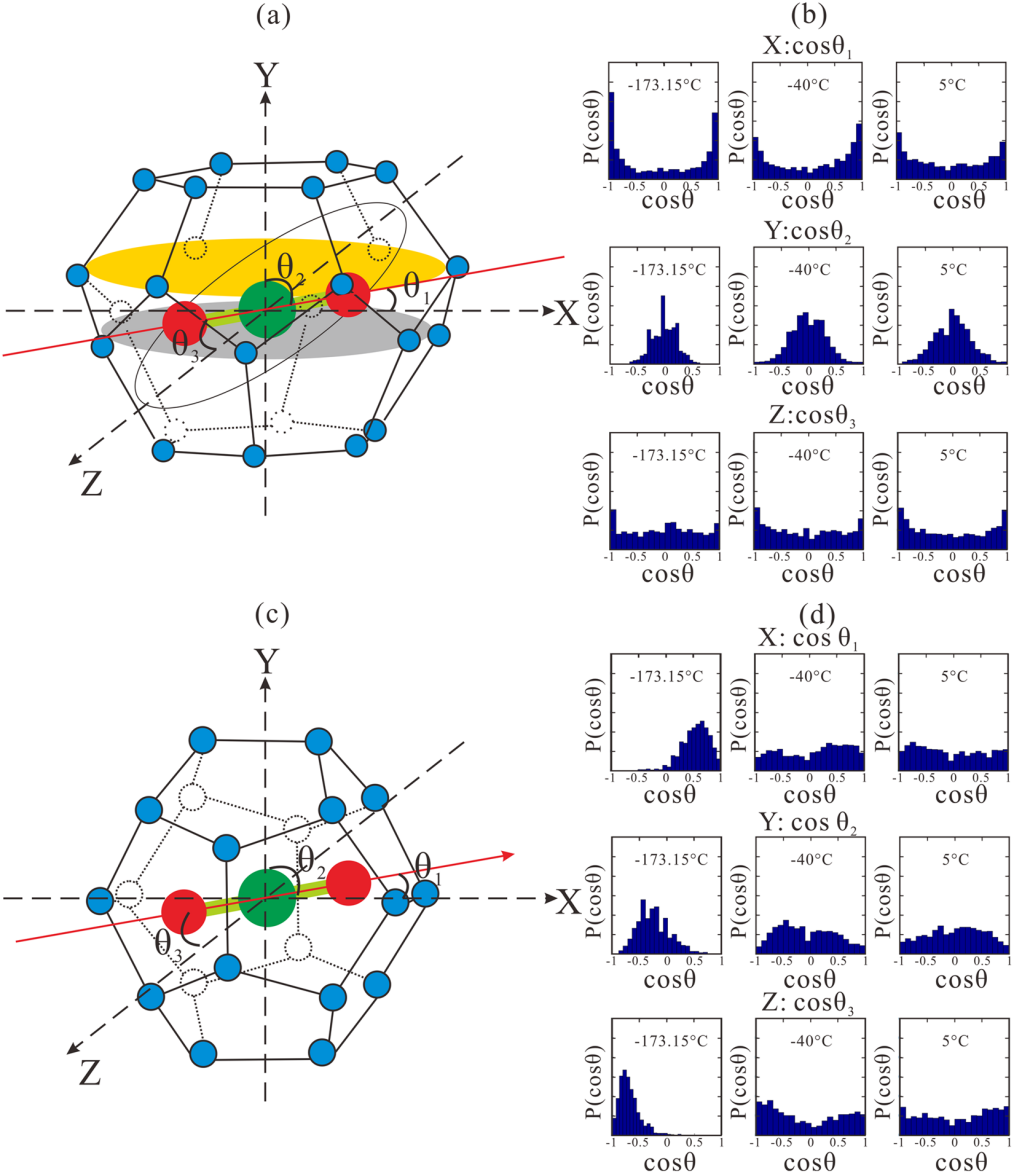
Kinetic behavior of CO_2_ molecules residing in L-cage (No. 1) and S-cage (No. 7), the equatorial plane in L-cage is perpendicular to Y axis. (**a**,**c**) Schematic graph of rotational motion of CO_2_ molecule. Red straight line represents the long axis of the CO_2_ molecule. Blue solid circles denote water molecules on the vertices of the cage. Red solid circles (oxygen atoms) and large green solid circle (carbon atom) comprise CO_2_ molecule. (**b**,**d**) Distribution of rotational correlation coefficients $(\cos \,\theta )$ in different directions for CO_2_ molecules shown in (**a**,**c**).

We have analyzed the rotational motions of all CO_2_ molecules from one primitive unit cell. According to the orientation of equatorial planes, the L-cages can be classified into: (1) the ones with equatorial planes which are perpendicular to Y axis are termed No. 1 and 2, that is, Y axis is the polar direction; (2) the ones with equatorial planes that are perpendicular to Z axis are termed No. 3 and 4, that is, Z axis is the polar direction; and (3) those with equatorial planes which are perpendicular to X axis are termed No. 5 and 6, that is, X axis is the polar direction. The two S-cages are termed No. 7 and 8. We take No. 1 L-cage (Fig. 4(a)) and No. 7 S-cage (Fig. 4(c)) for examples, where ${\theta }_{1}$ represents the angle between the X axis and long axis of the CO_2_ molecule (red line). ${\theta }_{2}$ denotes the angle between the Y axis and the red line, ${\theta }_{3}$ denotes the angle between the Z axis and the red line. Figure 4(b) and (d) shows the distributions of these angles at 40 MPa with varying temperatures in the three directions respectively. In addition to the P–T conditions within the range of diagrams, an extra data point (−173.15 °C, 40 MPa) is calculated to facilitate our discussions below.

Regarding No. 1 L-cage from the middle centering distributions of the cosine value of ${\theta }_{2}$, we know that most of the angles are close to 90°, which indicates that the CO_2_ molecule’s long axis is easier to rotate within a confined area in the vicinity of the equatorial plane because the direction of the symmetry axis is too short for full rotational motion (Fig. 4(a)). When the temperature increases, the orientation range of CO_2_ molecule is enlarged. This happens because the thermal-enhanced kinetic energy of the CO_2_ molecule can overcome the restrictions induced by the aspherical cavity and enable more rotational motions near the short symmetry axis direction.

Interestingly, the angle distribution of ${\theta }_{1}$ appears similar to that of ${\theta }_{3}$, which implies resembling behaviors. The CO_2_ molecule rotates mainly about the short symmetry axis near the equatorial plane. The projection of the distribution probability on the X–Z plane is uniform. ${\theta }_{1}$ and ${\theta }_{3}$ can be resolved into motions on the X–Y plane in conjunction with the X–Z and Y–Z plane in conjunction with X–Z plane, respectively. The X–Y component and Y–Z component are concentrated near the equatorial plane, which induces the same “saddle” shapes of combined motion with regard to $\cos \,{\theta }_{1}$ and $\cos \,{\theta }_{3}$. In supplementary materials, the distributions of ${\theta }_{1}$, ${\theta }_{2}$, and ${\theta }_{3}\,$with regard to the other two molecules (in L-Cages of No. 3 and No. 5) from the same primitive unit cell are documented. Concerning No. 3 L-cage (Fig. S3) and No. 5 L-cage (Fig. S4) molecules, the angles between the polar direction and the long axis of CO_2_ molecule are middle centering at 90° under low temperature. When the temperature increases to 5 °C, the distribution of cosine values of polar angles further widen to 1 or −1 implying the rotational plane can be along the short symmetry axis of the L-cages. All distribution patterns in L-cages indicate that the molecules rotate about the short symmetry axis near the equatorial plane and the thermal effect can enable more uniform angle distributions (See Figs 4(a) and (b), S3–S4). For the S-cages (Fig. 4(c) and (d)), the distribution of CO_2_ rotational motion was “locked” within a narrow range at low temperature and become uniform as the temperature increases. That is, the temperature significantly influenced the rotational motions of CO_2_ molecules in S-cages. Hence these temperature-enhanced freedoms of rotational motions make the entire structure more stable and more rigid.

Besides, two parallel layers exist with the same longest radii at the equatorial plane. One is composed of upper water molecules of the zigzag structure in the middle (orange ellipse in Fig. 4); the other exists along the bottom water molecules of the zigzag structure (grey ellipse in Fig. 4). Thus, the centroid of the CO_2_ molecule can move between the two layers to gain the largest freedom of rotational motion. Here, thermal expansion may be an important effect with temperature increasing. At 40 MPa, for example, the unit cell lengths are 1.182 nm, 1.196 nm, and 1.201 nm at −173.15 °C, −40 °C, and 5 °C respectively. As the space inside the cages increases with the increase in temperature, the CO_2_ molecule moves more easily and rotational motion becomes easier compared with the situation at low temperature. That is, expanded cages facilitate guest CO_2_ rotational motions, which enhance the stability of CO_2_ hydrate. In conclusion, the thermal effect is a critical external factor that plays an important role in affecting the stability and rigidity of the CO_2_ hydrate.

To corroborate that the observed anomaly indeed results from CO_2_ guest molecules, we have also investigated the elastic constants of a hypothetical hydrate structure without “guest” molecules (Fig. S5). The result shows that the variation trends of *C*_11_ and *C*_12_ are same to those of CH_4_ and CO_2_ hydrate, however, *C*_44_ of empty cage structure decreases regularly (like CH_4_ hydrate) with the increase in temperature. This finding further demonstrates that the anomalous behavior of *C*_44_ and shear modulus (as well as Young modulus) of CO_2_ hydrate is due to the “entrapped” CO_2_ molecule.

Previous studies have shown that mechanical properties are susceptible to the cage occupancy^40, 47^. Since the occupancy of CO_2_ hydrate is likely a function of the synthesis pressure, it is interesting to discuss how the occupancy (especially, of the small cages) affects the above results (i.e. the anomalous behavior of *C*_44_ and shear modulus). For this purpose, 75% (both of the two S-cages are empty in the primitive unit cell) and 87.5% (one of the S-cage is empty in the primitive unit cell) occupancy were investigated by the same method, and the comparisons of elastic constants are shown in Figs S2 and S6. Elastic constant of *C*_44_ is nearly invariable with temperature increasing when no CO_2_ molecules exist in the S-cages, and dramatically increases when half of the S-cages are occupied by CO_2_ molecules. We conclude that the CO_2_ molecules existing in the S-cages play a decisive role in elevating the stability and rigidity of the material with temperature increasing. For the most recent experiments, where the S-cage occupancy was found to be ~2/3^36, 37^, we anticipate that CO_2_ hydrate exhibits the anomalous thermally-enhanced-stability and -rigidity phenomenon, since the occupancy is indeed larger than 87.5% occupancy case (half in terms of S-cage occupancy).

## Discussions

In solid state physics, Born criterion^54, 55^ has been widely used to address the mechanical stability of a crystalline structure^48^. The violation of the Born criterion is regarded as the mechanism causing the onset of the structural transformation, including melting, polymorphism, and pressure-induced amorphization^48, 54–57^. For a cubic system, three conditions need to be fulfilled: ${C}_{44} > 0$, ${C}_{11}-{C}_{12} > 0$, and *C*_11_ + 2*C*_12_ > 0. The vanishing of *C*_44_ and tetragonal shear $({C}_{11}-{C}_{12})$ was suggested to be responsible for melting^54, 56^. For normal crystalline materials, as the temperature increases, the shear modulus presents softening behavior and eventually leads to a collapse of the crystal lattice into a liquid phase (with a shear modulus equal to zero). However, we show here that the shear modulus of CO_2_ hydrate increases with the temperature increasing. On the other hand, according to thermodynamic data, CO_2_ hydrate decomposes into ice and CO_2_ dry ice at around −150 °C^12, 30–32^. It is shown here that the shear modulus indeed decreases with the temperature decreasing. When the temperature is lower than −120 °C, the calculated stress-strain curves for *C*_44_ clearly display non-linear behaviors regardless of strain rate (Fig. S7) which implies an instability of the crystalline structure of CO_2_ hydrate. We show that this instability may result from the “locked” rotational motions of CO_2_ molecules, in particular, those in small cages, under low-temperature condition. When the small cages are empty, the calculated stress-strain curves for *C*_44_ clearly display linear again (Fig. S7). This indicates that the stability can be tuned by the occupancy of small cages. So far, the experimental evidence of CO_2_ hydrate decomposition at low-temperature limit has been indirect, namely by observing the pressure hysteresis by cooling the CO_2_ hydrate to −168.15 °C (105 K) and warming to around −43.15 °C (230 K)^30^. Structural analyses^37^ are needed for CO_2_ hydrate (presumably with high CO_2_ occupancy) to study the above-documented phase changes in detail. This study provides a new perspective on gas hydrates and new physics on temperature-enhanced stability of a crystalline material. Finally, elasticity anomaly may be anticipated in materials with the negative thermal expansion coefficient, as an example, the shear modulus was found to be increased slightly with temperature in the high-temperature β-quartz^58^. The anharmonic atomistic motions are crucial to describe the high-temperature elasticity behavior, and yet to be explored.

## Methods

### Main theory applied for mechanical properties

The generalized Hooke’s Law^51^ was used to investigate the stress–strain relationships of the gas hydrate, which is a cubic system and has only three independent elastic constants, namely *C*_11_, *C*_12_, and *C*_44_. The constitutive equation could be expressed as:

1 $$(\begin{array}{c}{\sigma }_{1}\\ {\sigma }_{2}\\ {\sigma }_{3}\\ {\sigma }_{4}\\ {\sigma }_{5}\\ {\sigma }_{6}\end{array})=(\begin{array}{llllll}{C}_{11} & {C}_{12} & {C}_{12} & 0 & 0 & 0\\ {C}_{12} & {C}_{11} & {C}_{12} & 0 & 0 & 0\\ {C}_{12} & {C}_{12} & {C}_{11} & 0 & 0 & 0\\ 0 & 0 & 0 & {C}_{44} & 0 & 0\\ 0 & 0 & 0 & 0 & {C}_{44} & 0\\ 0 & 0 & 0 & 0 & 0 & {C}_{44}\end{array})(\begin{array}{l}{\varepsilon }_{1}\\ {\varepsilon }_{2}\\ {\varepsilon }_{3}\\ {\varepsilon }_{4}\\ {\varepsilon }_{5}\\ {\varepsilon }_{6}\end{array})$$

where ${\sigma }_{{\rm{i}}}$ and ${\varepsilon }_{{\rm{i}}}$ represent the stress and strain tensors, respectively. *C*_ij_ is the elasticity matrix of materials, which determines the stiffness. The subscripts (1, 2, 3, 4, 5, 6) denote different directions (XX, YY, ZZ, ZY, ZX, YX). The elastic constants are the ratio of stress to corresponding strain and can be obtained from the slope of stress–strain curves if the deformation exists in the elastic regime. It is anticipated that the microscopic method (MD simulations) reflects the experimental observations well within the elastic regime of stress-strain relationships. Once the elastic constants are derived, the bulk modulus *K* and shear modulus *G* are evaluated using the Voigt model and assuming that strain is uniform and the Reuss model^52, 53^ by considering that the stress is uniform throughout the system.

Voigt model:

2 $${K}_{{\rm{V}}}=\frac{1}{3}{C}_{11}+\frac{2}{3}{C}_{12}$$

3 $${G}_{{\rm{V}}}=\frac{1}{5}{C}_{11}-\frac{1}{5}{C}_{12}+\frac{3}{5}{C}_{44}$$

Reuss model:

4 $$\frac{1}{{K}_{{\rm{R}}}}=3{S}_{11}+6{S}_{12}$$

5 $$\frac{1}{{G}_{{\rm{R}}}}=\frac{4}{5}{S}_{11}-\frac{4}{5}{S}_{12}+\frac{3}{5}{S}_{44}$$

where ${S}_{{\rm{ij}}}$ represents the compliance matrix, which is the reverse matrix of the elasticity matrix *C*_ij_. Since the Voigt and Reuss models signify the maximum and minimum value of the moduli, respectively, the results are optimized by the Hill average^52, 53^:

6 $$K=\frac{{K}_{{\rm{V}}}+{K}_{{\rm{R}}}}{2}$$

7 $$G=\frac{{G}_{{\rm{V}}}+{G}_{{\rm{R}}}}{2}$$

The results of the Hill average are used as input to evaluate Young’s modulus *E* and Poisson’s ratio *v* as follows:

8 $$E=\frac{9KG}{3K+G}$$

9 $$\nu =\frac{3K-2G}{2(3K+G)}$$

The P-wave velocity and S-wave velocity are calculated using bulk modulus *K* and shear modulus *G* as follows,

10 $${V}_{{\rm{P}}}=\sqrt{\frac{K+\frac{4}{3}G}{\rho }}$$

11 $${V}_{{\rm{S}}}=\sqrt{\frac{G}{\rho }}$$

*ρ* denotes density of the system.

### Simulation details

The GROMACS version 4.5.5^59^ was used to perform the equilibrium and non-equilibrium MD simulations in this study. A Nosé–Hoover thermostat^60^ and Parrinello–Rahman pressure coupling^61^ were used for temperature and pressure control, respectively. A particle mesh Ewald summation method^62^ was used to calculate long-range electrostatic interactions. The cutoff distance for Van der Waals interactions was set to 1.1 nm. A time step of 1 fs was set to integrate the motion equations with the leapfrog algorithm^63^.

The TIP4P/Ice^64, 65^ OPLS_AA^66, 67^, and EPM2^68^ models (potential parameters in the Table SIII) were used to model behaviors of water, CH_4_, and CO_2_ molecules in the simulations. For CO_2_, TraPPE^69^ model was also employed to test whether the conclusion is dependent on the model that we used. While most of our results for CO_2_ hydrate are based on EPM2 model, the result from TraPPE is presented in Fig. S2. Previous studies have shown that the models describe the liquid-solid phase transition of ice Ih, and three-phase coexistence line of CH_4_ hydrates very well^64, 65^. In addition, our preliminary studies using the same model could reproduce three-phase coexistence line of carbon dioxide hydrates. We used the proton-disordered unit cell of gas hydrate (sI) with a vanishingly small total dipole moment^45, 48^. The unit cell was duplicated six times in each direction to obtain the initial configurations. This structure consists of 1728 “guest” molecules and 9936 water molecules. Initially, all configuration boxes were equilibrated in an isothermal–isobaric ensemble for 1 ns under the required temperature and pressure. To calculate the elastic constants, a constant compressive and tensile strain were applied up to 0.04 in the XX direction and shear strains (both positive and negative directions) were applied up to 0.1 in the ZX direction. We used strain rates of 4 × 10^8^ s^−1^ and 1 × 10^9^ s^−1^ for uniaxial strain and shear strain when calculating the elastic moduli, respectively. Additional studies show that the elastic constants are independent of the loading speeds within the elastic regime^46^. Five independent simulation runs were performed for CO_2_ hydrate under 40 MPa and 0 °C in order to investigate the calculation reliability, and the results are given in Table SIV. Furthermore, similar calculations were performed at 10 different temperatures under 40 MPa. Calculated *C*_11_, *C*_12_, and *C*_44_ were shown in Fig. S8. The standard deviation of each parameter is at least ten times less than difference between the values of −40 °C and 5 °C with same pressure, which indicates the variation trends (as shown in Figs 2 and 3) are not affected by calculation uncertainty. This is indeed the case as presented in Fig. S8.

## Electronic supplementary material

Supplementary Materials
