# Supplementary material for: Elasticity and Stability of Clathrate Hydrate: Role of Guest Molecule Motions
Source: Sci Rep. 2017 May 2;7:1290. doi: 10.1038/s41598-017-01369-0 (PMC5431056; doi:10.1038/s41598-017-01369-0)
Supplement: Supplementary file 1 — Supplementary Materials [file 41598_2017_1369_MOESM1_ESM.pdf]

Supplementary Materials

Elasticity and Stability of Clathrate Hydrate: Role of Guest Molecule Motions

Jihui Jia,[1,2] *Yunfeng Liang,*[1,3*] *Takeshi Tsuji,*[2,4] *Sumihiko Murata,*[1*] *and Toshifumi Matsuoka*[1,5]

[1]*Environment and Resource System Engineering, Kyoto University, Kyoto 615-8540, Japan*

[2]*International Institute for Carbon-Neutral Energy Research (I2CNER), Kyushu University, Fukuoka 819-0395, Japan*

[3]*Center for Engineering, Research into Artifacts (RACE), the University of Tokyo, Chiba 277-8568, Japan*

[4]*Department of Earth Resources Engineering, Kyushu University, Fukuoka 819-0395, Japan*

[5]*Fukada Geological Institute, Tokyo 113-0021, Japan*

[*]Corresponding author: Yunfeng Liang

Postal address: The University of Tokyo
Room 566, Kashiwa Research Complex 5F, Kashiwa Campus
5-1-5 Kashiwanoha, Kashiwa
Chiba 277-8568, Japan

Tel: +81-3-7136-4271

Email: liang@race.u-tokyo.ac.jp

*Corresponding author: Sumihiko Murata
Postal address: Kyoto University
Room C1-1-108
Kyotodaigaku-Katsura, Nishikyoku
Kyoto 615-8540, Japan
Tel: +81-75-383-3204
Email: murata.sumihiko.6v@kyoto-u.ac.jp

Supplementary Results

The following supplementary materials are prepared for the article:

Figure S1. Calculated stress–strain curves for CH_4 and CO_2 hydrates at one pressure-temperature data point. They show good linear relationships within elastic regime. C_{11} and C_{12} are from same axial strain deformation, while C_{44} is from shear strain deformation.

Figure S2. Calculated elastic constants for CO_2 hydrate with different occupancy using TraPPE model. The dash lines of C_{44} panel are the fitted curves to the calculated data.

Figure S3. Kinetic behavior of CO_2 molecule residing in No.3 L-cage, indicating rotational motion of "entrapped" CO_2 molecule is near the equatorial plane of L-cage.

Figure S4. Kinetic behavior of CO_2 molecule residing in No.5 L-cage, indicating rotational motion of "entrapped" CO_2 molecule is near the equatorial plane of L-cage.

Figure S5. Diagram of elastic constants of a hypothetical hydrate structure without guest molecules. The range of temperature and pressure are same as Figs. 2 and 3. The C_{11}, C_{12} and C_{44} of no "guest" hydrate have same variation trend with CH_4 hydrate.

Figure S6. Three independent elastic constants of CO_2 hydrate with partial occupancy under conditions from −40°C to 5°C and 20 MPa to 110 MPa. 75% means no "guest" molecules existing in small cages. 87.5% represents that half of the small cages are occupied by "guest" molecules.

Figure S7. Calculated stress–strain curves of CO_2 hydrate with constant strain rate deformation simulations at 100 K and 20 MPa.

Figure S8. Three independent elastic constants as function of temperature for fully occupied CO_2 hydrate under conditions from −40°C to 5°C and at 40 MPa. The results show that the calculation uncertainty is small enough and would not influence the observed variation trend of elastic constants and moduli.

Tab. SI. (a) Lattice constants of different gas hydrates under 0.1 MPa with temperatures from P-T diagrams, the unit is nanometers. (b) Thermal expansivity regarding lattice constants for different gas hydrates.

Tab. SII. Lattice constants of different gas hydrates with pressure of 0.1 MPa under 200 K.

Tab. SIII Lennard-Jones potential parameter ε and σ, partial charges q, and geometry of the H_2O, CH_4 and CO_2 models used in this work.

Tab. SIV. Results of five independent calculations for fully occupied CO_2 hydrate under 40 MPa and 0°C. The results show that the calculation uncertainty is small enough and would not influence the observed variation trend of elastic constants and moduli.

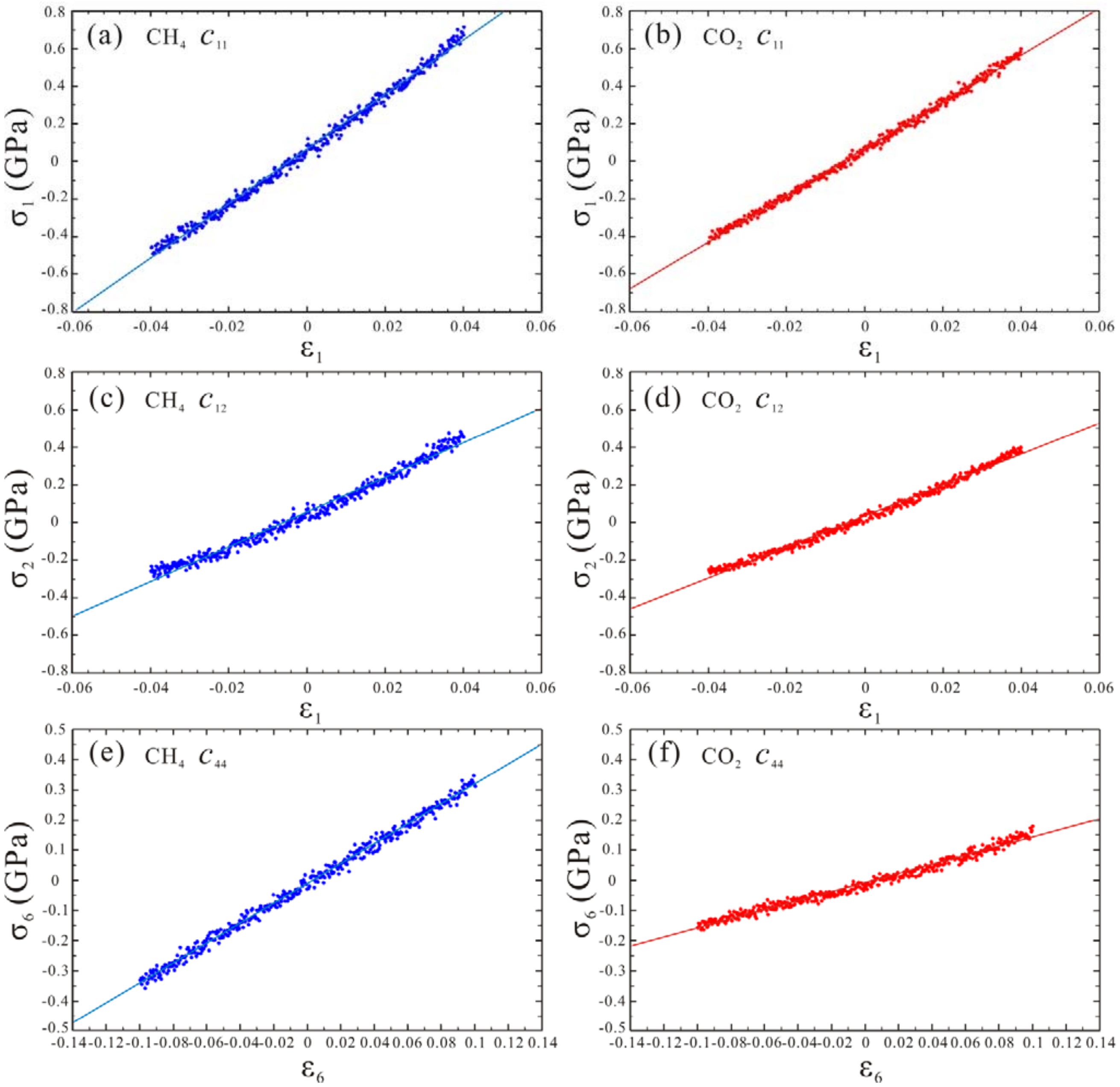

Figure S1. Stress-strain behaviors of gas hydrate with constant strain rate deformation simulations. Calculated stress–strain curves for CH_4 (a, c, e) and CO_2 (b, d, f) hydrates. C_{11} and C_{12} are from the same axial deformation, C_{44} is from the shear deformation.

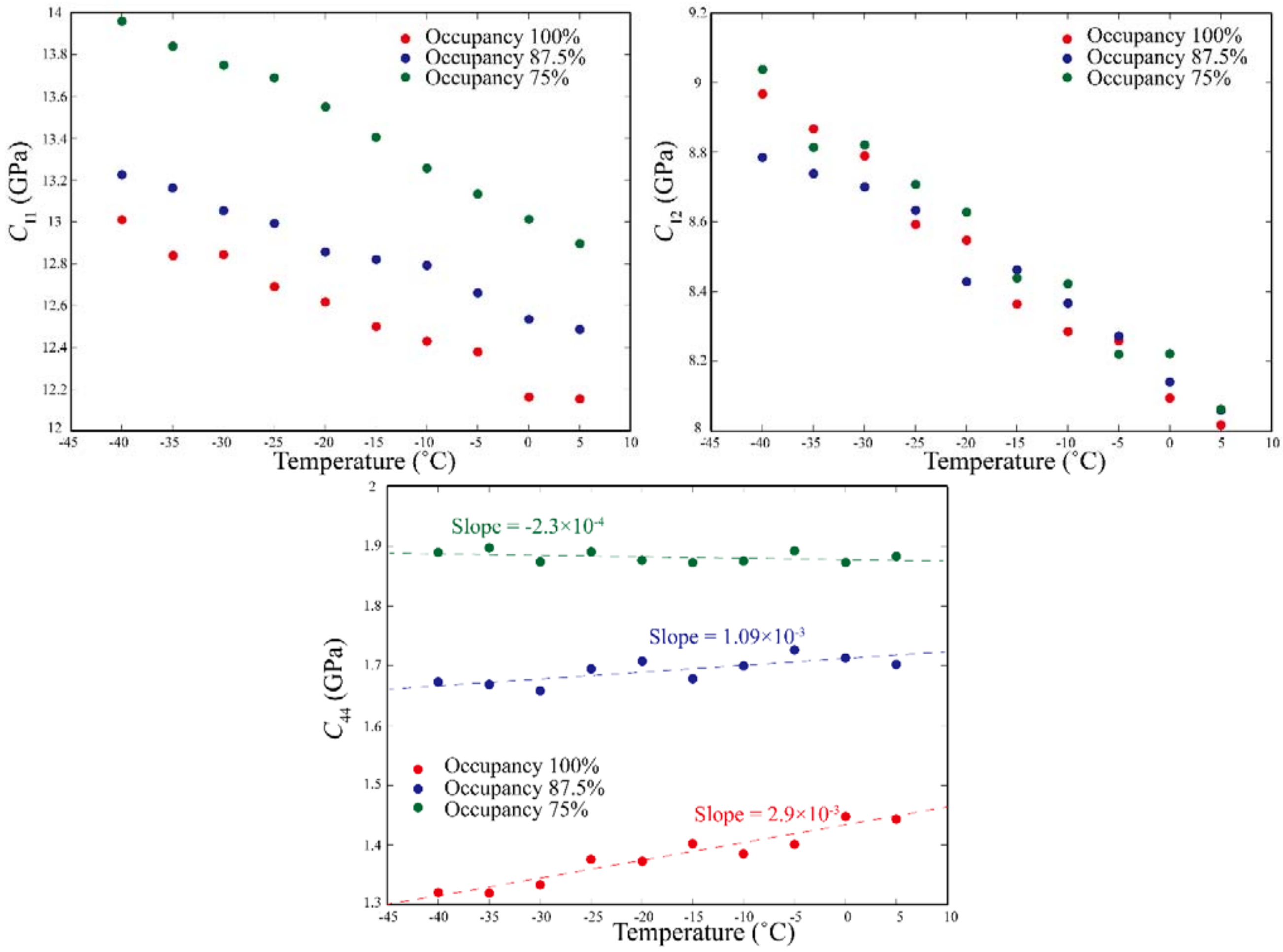

Figure S2. Calculated elastic constants for CO_2 hydrate with different occupancy at 40 MPa and at elevated temperatures using TraPPE model. The dash lines of C_{44} panel are the fitted curves to the calculated data. The slopes of full occupancy and 87.5% occupancy are positive, whereas that of 75% occupancy the slope is negative. The absolute values differ by one order magnitude.

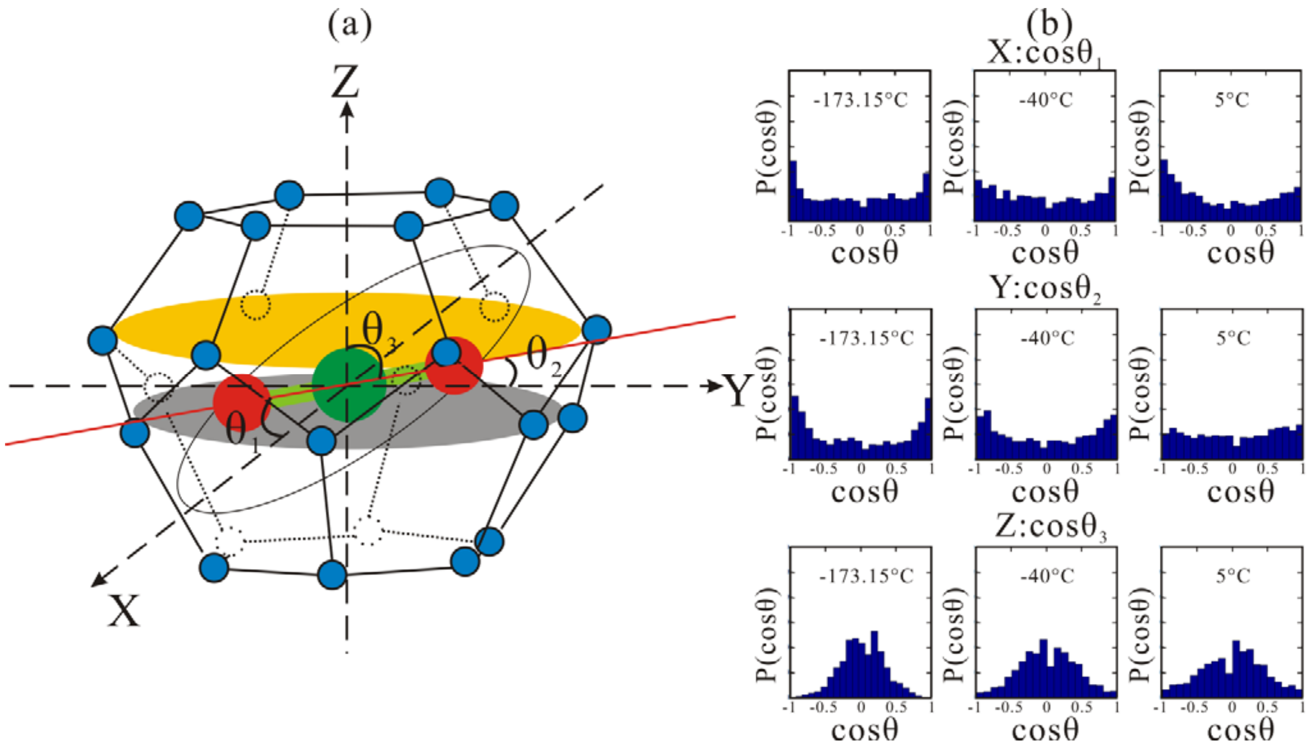

Figure S3. Kinetic behavior of CO_2 molecule residing in No.3 L-cage, the equatorial plane is perpendicular to Z axis (note: No.4 L-cage has the same orientation). (a) Schematic graph of rotational motion of CO_2 molecule. Red straight line represents the long axis of the CO_2 molecule. Blue solid circles denote water molecules on the vertices of the cage. Red solid circles (oxygen atoms) and large green solid circle (carbon atom) comprise CO_2 molecule. (b) Distribution of rotational correlation coefficients ($\cos\theta$) in different directions for CO_2 molecule shown in Panel (a).

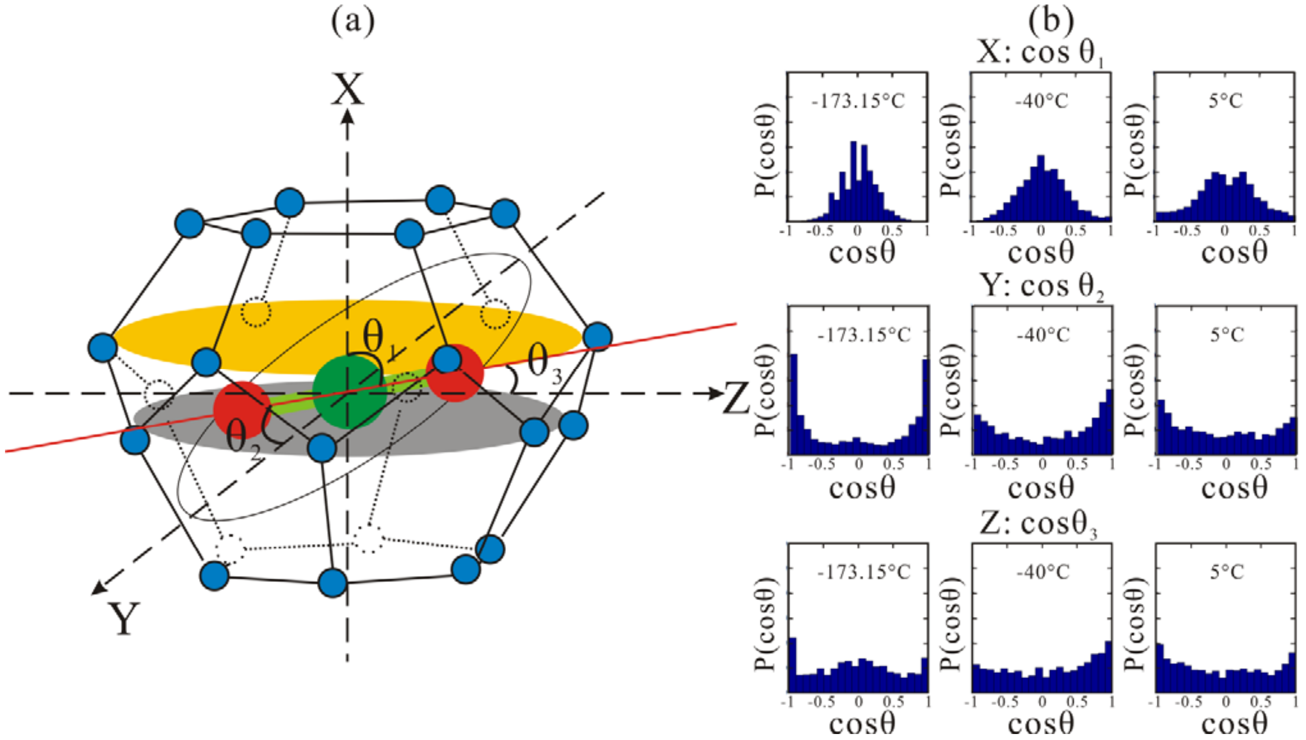

Figure S4. Kinetic behavior of CO_2 molecule residing in No.5 L-cage, the equatorial plane is perpendicular to X axis (note: No.6 L-cage has the same orientation). (a) Schematic graph of rotational motion of CO_2 molecule. Red straight line represents the long axis of the CO_2 molecule. Blue solid circles denote water molecules on the vertices of the cage. Red solid circles (oxygen atoms) and large green solid circle (carbon atom) comprise CO_2 molecule. (b) Distribution of rotational correlation coefficients ($\cos\theta$) in different directions for CO_2 molecule shown in Panel (a).

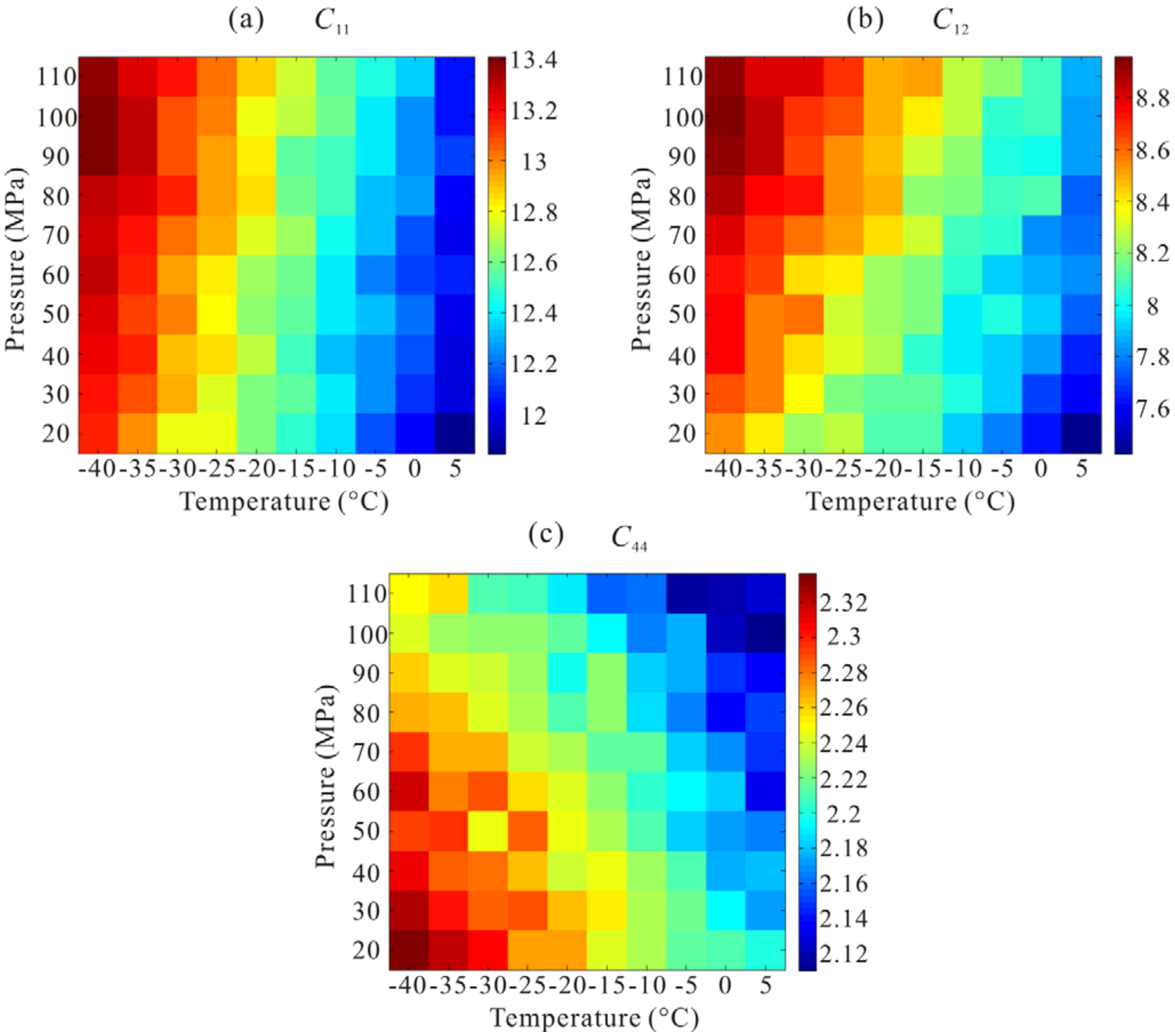

Figure S5. Three independent elastic constants of the hypothetical hydrate structure without "entrapped" guest molecules under conditions from −40°C to 5°C and 20 MPa to 110 MPa. Red represents large value while blue represents small value with unit of GPa.

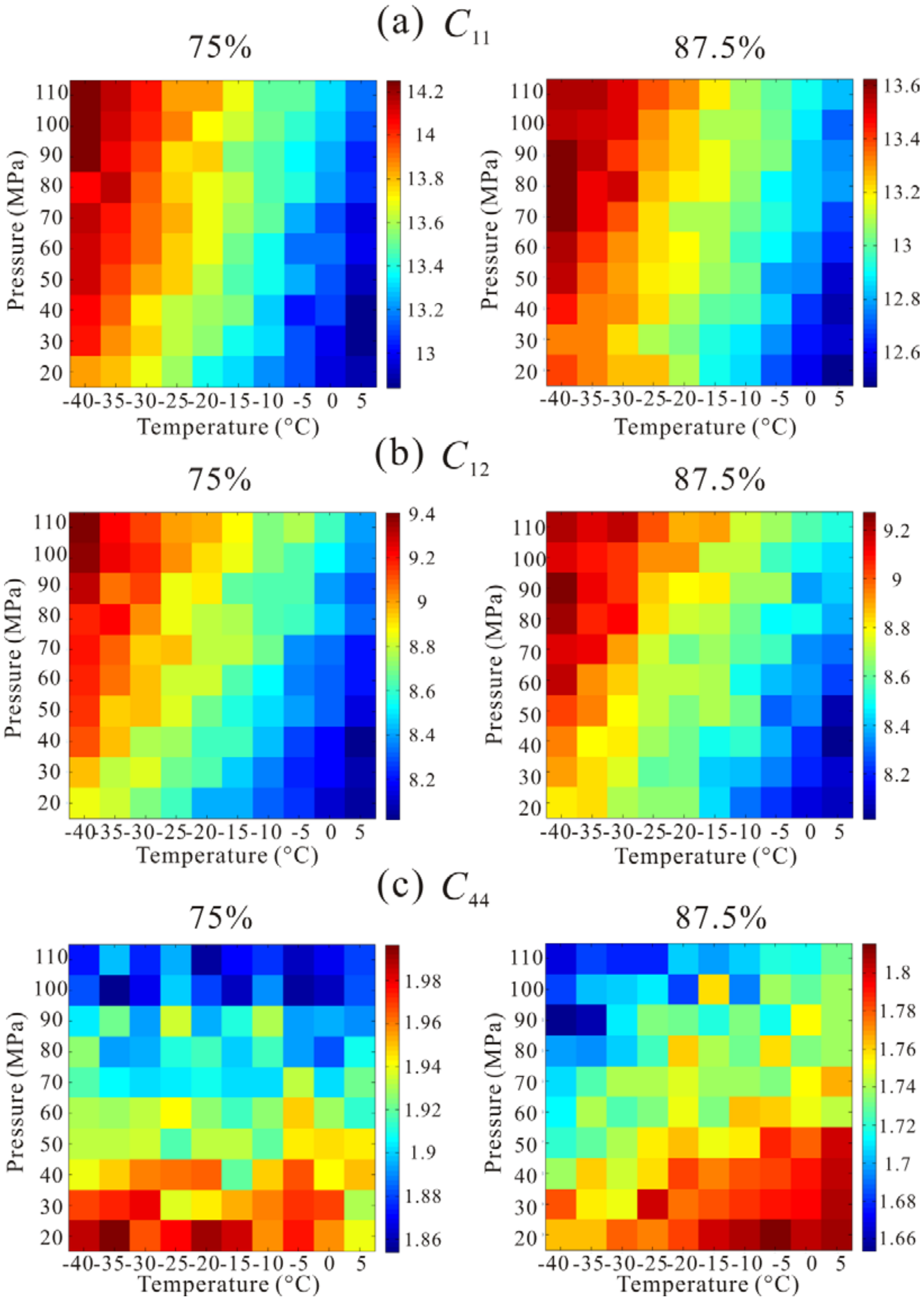

Figure S6. Three independent elastic constants of CO_2 hydrate with partial occupancy under conditions from −40°C to 5°C and 20 MPa to 110 MPa. 75% means no "guest" molecules existing in small cages. 87.5% represents that half of the small cages are occupied by "guest" molecules.

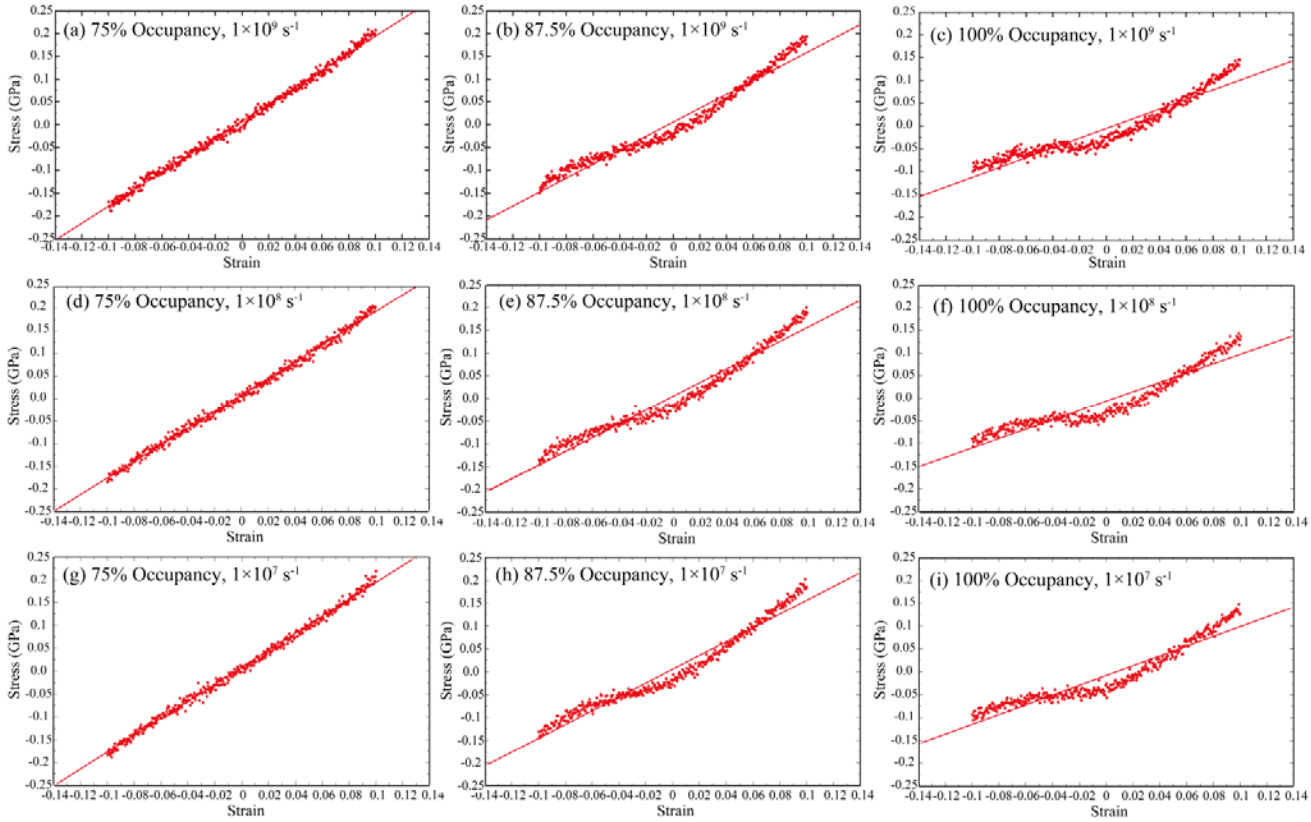

Figure S7. Calculated stress–strain curves of CO_2 hydrate with constant strain rate deformation simulations at 100 K and 20 MPa. (a) empty small cages (75%) with strain rate of 1×10^9 s^{-1}; (b) half-occupied small cages (87.5%) with strain rate of 1×10^9 s^{-1}; (c) fully-occupied cages (100%) with strain rate of 1×10^9 s^{-1}; (d) empty small cages (75%) with strain rate of 1×10^8 s^{-1}; (e) half-occupied small cages (87.5%) with strain rate of 1×10^8 s^{-1}; (f) fully-occupied cages (100%) with strain rate of 1×10^8 s^{-1}; (g) empty small cages (75%) with strain rate of 1×10^7 s^{-1}; (h) half-occupied small cages (87.5%) with strain rate of 1×10^7 s^{-1}; (i) fully-occupied cages (100%) with strain rate of 1×10^7 s^{-1}.

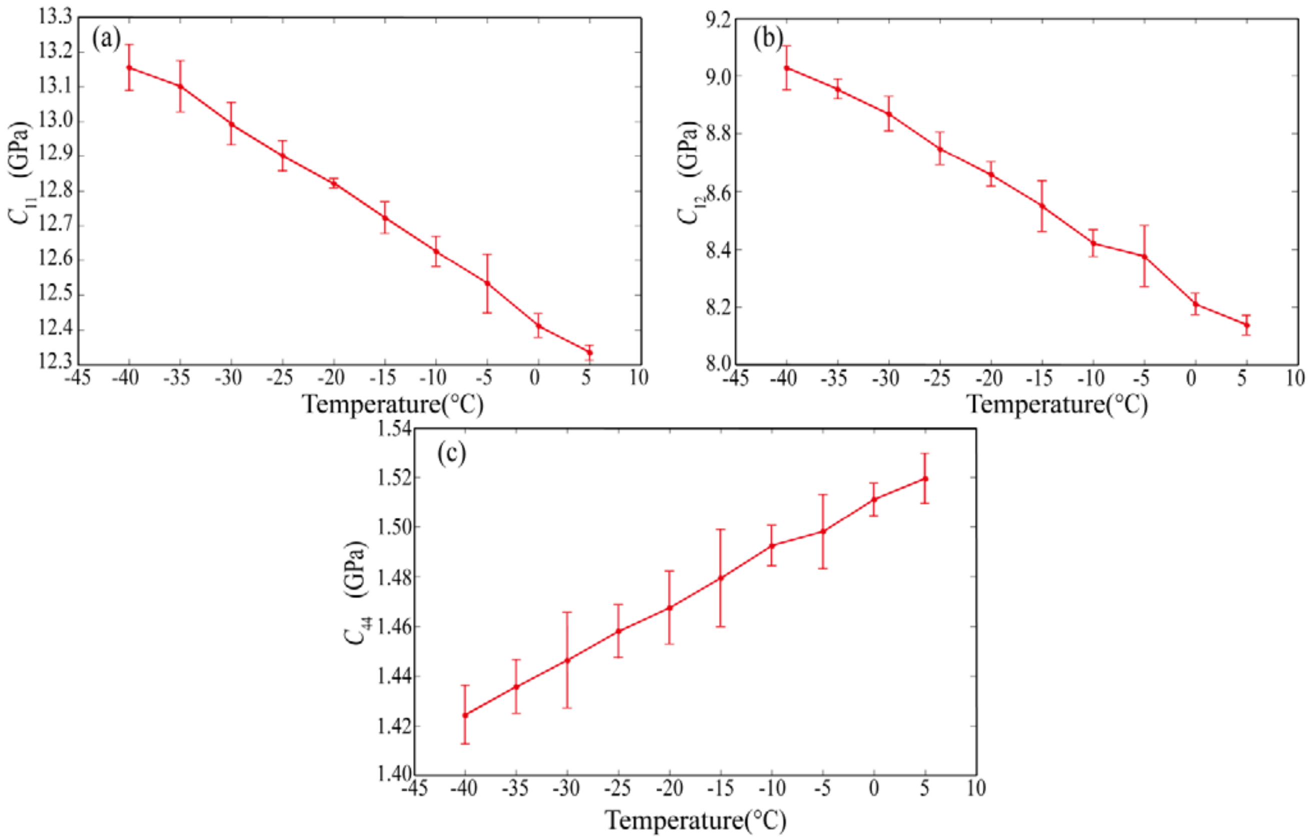

Figure S8. Three independent elastic constants (C_{11}, C_{12}, and C_{44}) as function of temperature for fully occupied CO_2 hydrate under conditions from −40°C to 5°C and at 40 MPa. The standard deviation is obtained on the basis of five independent calculations.

Table SⅠ. (a) Lattice constants of different gas hydrates at 0.1 MPa with temperature range of P-T diagrams, the unit is nanometers. (b) Thermal expansivity regarding lattice constants for different gas hydrates, delta (Δ) denotes the difference of lattice constants values between 233.15 K and 278.15 K, which is divided by the former. Experimental data is from Hansen *et al.* 2016[1]. *Est* means the estimated value for CO_2 hydrate in which small cage occupancy is 2/3, by assuming that the increment is based on equal ratio. The deviation of CO_2 hydrate is defined by the relative difference between the estimated values and the experiments[1]. The deviation of CH_4 hydrate is defined by the relative difference between the calculated values (with 100 % occupancy) and the experiments[1].

T	233.15K	238.15K	243.15K	248.15K	253.15K	258.15K	263.15K	268.15K	273.15K	278.15K
Empty	1.1965	1.1969	1.1973	1.1976	1.1979	1.1982	1.1985	1.1990	1.1994	1.1997
CO_2_75	1.1951	1.1956	1.1960	1.1966	1.1970	1.1974	1.1980	1.1983	1.1989	1.1994
CO_2_87.5	1.1965	1.1969	1.1974	1.1980	1.1985	1.1989	1.1994	1.2000	1.2006	1.2010
CO_2_100	1.1976	1.1982	1.1988	1.1993	1.1998	1.2004	1.2009	1.2016	1.2020	1.2026
CO_2_Est	1.1967	1.1971	1.1976	1.1982	1.1987	1.1991	1.1997	1.2003	1.2008	1.2013
CH_4_100	1.1963	1.1968	1.1974	1.1979	1.1981	1.1988	1.1992	1.1996	1.2001	1.2007
CO_2_Exp	1.1938	1.1943	1.1948	1.1952	1.1957	1.1961	1.1967	1.1971	1.1977	1.1983
CH_4_Exp	1.1934	1.1939	1.1943	1.1948	1.1951	1.1957	1.1962	1.1967	1.1971	1.1976
Deviation_CO_2	0.0024	0.0023	0.0023	0.0025	0.0025	0.0025	0.0025	0.0027	0.0026	0.0025
Deviation_CH_4	0.0024	0.0024	0.0026	0.0026	0.0025	0.0026	0.0025	0.0024	0.0025	0.0026

T	Δ(×10^{-3})
Empty	2.620
CO_2_75	3.521
CO_2_87.5	3.762
CO_2_100	4.242
CO_2_Est	3.842
CH_4_100	3.638
CO_2_Exp	3.770
CH_4_Exp	3.519

Table SⅡ. Lattice constants of different gas hydrates with pressure of 0.1 MPa under 200 K. The deviation is defined in Table SI.

T	100K	120K	140K	160K	180K	200K
Empty	1.1886	1.1897	1.1908	1.1919	1.1931	1.1944
CO_2_75	1.1845	1.1859	1.1874	1.1889	1.1905	1.1922
CO_2_87.5	1.1843	1.1860	1.1877	1.1896	1.1914	1.1934
CO_2_100	1.1846	1.1864	1.1884	1.1903	1.1922	1.1942
CO_2_Est	1.1844	1.1861	1.1878	1.1897	1.1915	1.1935
CH_4_100	1.1862	1.1875	1.1889	1.1904	1.1919	1.1936
CO_2_Exp	1.1844	1.1854	1.1866	1.1879	1.1894	1.1909
CH_4_Exp	1.1850	1.1859	1.1868	1.188	1.1892	1.1907
Deviation_CO_2	0	0.0006	0.0010	0.0015	0.0018	0.0022
Deviation_CH_4	0.0010	0.0014	0.0018	0.0020	0.0023	0.0024

Table SIII Lennard-Jones potential parameters ε and σ , partial charges q, and geometry of the H_2O , CH_4 and CO_2 models used in this work.

Atom	ε (KJ/M)	σ (Å)	q (e)	Geometry
TIP4P/Ice H_2O				
O	0.8822	3.1668	0	O-H: 0.9572 Å
H	0	0	0.5879	O-M: 0.1546 Å
M	0	0	-1.1794	H-O-H: 104.52°
OPLS_AA CH_4				
C	0.2761	3.50	-0.24	C-O: 1.09 Å
H	0.1255	2.50	0.06	H-C-H: 109.47°
EPM2* CO_2				
C	0.2338	2.757	0.6512	C-O: 1.16 Å
O	0.6691	3.033	-0.3256	O-C-O: 180°
TraPPE CO_2				
C	0.2245	2.80	0.70	C-O: 1.16 Å
O	0.6568	3.05	-0.35	O-C-O: 180°

*The C-O bond is as long as TraPPE model which is consistent with experimental results.

Table SIV. Elastic constants and moduli from five independent calculations performed under pressure of 40 MPa and temperature of 0°C for CO_2 hydrate, maximum difference represents the difference between values of -40°C and 5°C with same Pressure of 40 MPa.

	C_{11}	C_{12}	C_{44}	K	G	E	ν
Run 1	12.4291	8.2134	1.5053	9.6186	1.723	4.8777	0.4155
Run 2	12.4242	8.2341	1.5042	9.6308	1.718	4.8647	0.4158
Run 3	12.4529	8.2421	1.5106	9.6457	1.7258	4.886	0.4156
Run 4	12.3748	8.1495	1.5195	9.5579	1.7342	4.906	0.4145
Run 5	12.3788	8.2134	1.5158	9.6019	1.7218	4.874	0.4154
Standard deviation	3.391×10^{-2}	3.638×10^{-2}	6.598×10^{-3}	3.375×10^{-2}	6.074×10^{-3}	1.56×10^{-2}	5.03×10^{-4}
Maximum difference	7.487×10^{-1}	8.674×10^{-1}	7.08×10^{-2}	8.278×10^{-1}	6.8×10^{-2}	1.61×10^{-1}	9.443×10^{-3}

Reference

1. Hansen, T. C., Falenty, A. & Kuhs, W. F. Lattice constants and expansivities of gas hydrates from 10 K up to the stability limit. *J. Chem. Phys.* **144**, 054301 (2016).
